# Human leptospirosis in Seychelles: A prospective study confirms the heavy burden of the disease but suggests that rats are not the main reservoir

**DOI:** 10.1371/journal.pntd.0005831

**Published:** 2017-08-28

**Authors:** Leon Biscornet, Koussay Dellagi, Frédéric Pagès, Jastin Bibi, Jeanine de Comarmond, Julien Mélade, Graham Govinden, Maria Tirant, Yann Gomard, Vanina Guernier, Erwan Lagadec, Jimmy Mélanie, Gérard Rocamora, Gildas Le Minter, Julien Jaubert, Patrick Mavingui, Pablo Tortosa

**Affiliations:** 1 Université de La Réunion, UMR PIMIT (Processus Infectieux en Milieu Insulaire Tropical), CNRS 9192, INSERM U 1187, IRD 249. Plateforme de Recherche CYROI, Sainte Clotilde, La Réunion, France; 2 CRVOI, Centre de Recherche et de Veille sur les Maladies Emergentes dans l’Océan Indien, Ste Clotilde, Plateforme de Recherche CYROI, Sainte Clotilde, La Réunion, France; 3 Infectious Disease Surveillance Unit, Seychelles Public Health Laboratory, Public Health Authority, Ministry of Health, Mont Fleuri, Seychelles; 4 Regional Office of the French Institute for Public Health Surveillance (Santé Publique France), Saint-Denis, La Réunion, France; 5 Disease Surveillance and Response Unit, Epidemiology and Statistics Section, Public Health Authority, Ministry of Health, Mont Fleuri, Seychelles; 6 Veterinary Services Section, Seychelles Agricultural Agency, Ministry of Agriculture and Fisheries, Victoria, Seychelles; 7 Island Biodiversity and Conservation Centre, University of Seychelles, Victoria, Seychelles; 8 Service de Bactériologie, Parasitologie, Virologie et Hygiène, Groupe Hospitalier Sud Réunion-Centre Hospitalier Universitaire (GHSR-CHU), Saint Pierre, La Réunion, France; Yale University Yale School of Public Health, UNITED STATES

## Abstract

**Background:**

Leptospirosis is a bacterial zoonosis caused by pathogenic *Leptospira* for which rats are considered as the main reservoir. Disease incidence is higher in tropical countries, especially in insular ecosystems. Our objectives were to determine the current burden of leptospirosis in Seychelles, a country ranking first worldwide according to historical data, to establish epidemiological links between animal reservoirs and human disease, and to identify drivers of transmission.

**Methods:**

A total of 223 patients with acute febrile symptoms of unknown origin were enrolled in a 12-months prospective study and tested for leptospirosis through real-time PCR, IgM ELISA and MAT. In addition, 739 rats trapped throughout the main island were investigated for *Leptospira* renal carriage. All molecularly confirmed positive samples were further genotyped.

**Results:**

A total of 51 patients fulfilled the biological criteria of acute leptospirosis, corresponding to an annual incidence of 54.6 (95% CI 40.7–71.8) per 100,000 inhabitants. *Leptospira* carriage in *Rattus* spp. was overall low (7.7%) but dramatically higher in *Rattus norvegicus* (52.9%) than in *Rattus rattus* (4.4%). *Leptospira interrogans* was the only detected species in both humans and rats, and was represented by three distinct Sequence Types (STs). Two were novel STs identified in two thirds of acute human cases while noteworthily absent from rats.

**Conclusions:**

This study shows that human leptospirosis still represents a heavy disease burden in Seychelles. Genotype data suggests that rats are actually not the main reservoir for human disease. We highlight a rather limited efficacy of preventive measures so far implemented in Seychelles. This could result from ineffective control measures of excreting animal populations, possibly due to a misidentification of the main contaminating reservoir(s). Altogether, presented data stimulate the exploration of alternative reservoir animal hosts.

## Author summary

Leptospirosis is an emerging environmental infectious disease caused by corkscrew shaped bacteria called *Leptospira*. Humans usually get infected during recreational or work-related outdoor activities through contact with urine excreted by animal reservoirs. As a zoonotic disease, leptospirosis is a good example of the One Health concept for it links humans, animals and ecosystems in a web of pathogen maintenance and transmission. This zoonosis is highly prevalent in the tropics and especially in tropical islands. Seychelles archipelago has been reported as the country with highest human incidence worldwide, although figures are based on dated studies and/or poorly specific tests. The presented investigation aimed at providing an updated information on human leptospirosis burden in Seychelles and exploring the transmission chains in their environmental aspects. Presented data confirms that the disease still heavily impacts the country. Genotyping of pathogenic *Leptospira* in human acute cases reveals that three distinct Sequence Types (STs) are involved in the disease. However, rats typically considered as the main reservoir in Seychelles, harbor only one of these STs, found only in a minority of human cases. Hence, it appears that rats are likely not the main reservoir of leptospirosis in Seychelles, which has important consequences in terms of preventive measures to be implemented for a better control of human leptospirosis.

## Introduction

Zoonoses are known to contribute to approximately 60% of human infectious diseases and represent 75% of emerging diseases [1]. Among them, leptospirosis is considered as one of the most prevalent bacterial zoonotic disease worldwide [2], as well as a (re)emerging disease [3]. It is considered by the WHO as being amongst the world’s neglected tropical diseases with epidemic-prone potential causing significant public health impact [4,5]. Leptospirosis affects 1.03 million persons annually causing nearly 60,000 deaths [6]. This zoonosis is transmitted to humans and domestic animals through direct or indirect contact with infected urine excreted by reservoir/carrier hosts [7]. Leptospirosis prevalence is higher in tropical environments where conditions may be more conducive to *Leptospira* survival [8]. Prevalence is maximal in tropical islands for unknown reasons, although reduced species diversity typical of insular ecosystems may boost pathogen transmission [9,10].

In the South West Indian Ocean islands (SWIO), human leptospirosis shows a contrasting epidemiology in terms of incidence, mortality and diversity of leptospires. In Reunion, a French administered island, the incidence of human leptospirosis is the lowest recorded in the region (8.2 cases per 100,000) and the disease is caused by two *Leptospira* species (*L*. *interrogans* and, to a much lesser extent, *L*. *borgpetersenii*), with Icterohaemorrhagiae serogroup being overwhelmingly dominant [11]. The higher incidence (74.5 cases per 100,000) in Mayotte [12], also administered by France and sharing a common health surveillance system with Reunion island, is compounded by a much higher diversity of bacterial species (*L*. *interrogans*, *L*. *borgpetersenii*, *L*. *kirschneri* and *L*. *mayottensis*) and serogroups [13,14]. Noteworthily in Mayotte, Icterohaemorrhagiae serogroup was not diagnosed through Microscopic Agglutination Test (MAT) since the implementation of active surveillance in 2008 [15]. In the Union of the Comoros, no human leptospirosis has been reported recently, but according to a recent study, the serological profile of *Leptospira* infecting humans is likely similar to that encountered in the neighboring Mayotte [16], the fourth island of the Comorian archipelago. In Madagascar, a considerable diversity of pathogenic *Leptospira* has been detected in bats and terrestrial small mammals [17–19], despite limited reports of human cases [20,21].

The Republic of Seychelles is an archipelago consisting of 155 granitic or coralline islands located between 4 and 10 degrees south of the equator and lying between 480 km and 1,600 km east of the African continent in the SWIO. The climate of Seychelles is of the warm, humid tropical type, and divided into two main seasons: the Northwest Monsoon, period of higher rainfall from December to March, and the Southeast Monsoon from May to October, separated by two relatively short Inter-Monsoonal periods [22]. The mid-year population estimate of Seychelles as at August 2015 is 93,419 [23], of which almost 90% live on Mahé island.

In reference studies on humans in Seychelles conducted up to 25 years ago [24–27], Seychelles has been reported as ranking first worldwide for leptospirosis incidence [8], and several serogroups have been identified by Microscopic Agglutination Test (MAT) [25,27]. However, in the absence of published molecular data, the *Leptospira* species of medical concern in Seychelles remains unknown. Rats have been considered as the main animal reservoir of *Leptospira* in Seychelles and have been the target of active population control; eradication of invasive rodents, including black rats (*Rattus rattus*) and Norway rats (*Rattus norvegicus*), have been a continuous activity of both the Public Health Authority and the Environment department in Seychelles [28]. However, the role of suspected animal reservoirs has never been properly investigated, and the only mention of *Leptospira* prevalence in rats is an anecdotal study where 24% of sampled rats (n = 25) were reported to be MAT seropositive for Icterohaemmorhagiae serogroup [25]. Hence, from a public health perspective, there is still a significant lack of information regarding a zoonosis considered as the most important infectious disease in the country, based on local surveillance data collected over the past three to four decades.

The context of insular countries, such as Seychelles, which inherently have limited geographical distribution and diversity of vectors/reservoirs, represents an opportune small-scale environmental setup for the investigation of disease ecology [29]. Such studies have a direct beneficial impact in providing concrete evidence-based data that may guide public health interventions implemented to control the transmission of zoonotic pathogens to humans. Hence, we carried out a comprehensive survey aiming at (i) determining the present incidence of human leptospirosis in Seychelles and compare it to the Yersin *et al*. (1998) study published almost twenty years ago, (ii) characterizing at the specific and infraspecific levels the *Leptospira* infecting humans and animal reservoirs in order to identify transmission chains, and (iii) identifying biotic and abiotic variables having a major impact on the epidemiology of the disease in Seychelles.

## Materials and methods

### Ethical approval and sampling permits

The Health Research and Ethics Committee of the Ministry of Health of Seychelles approved the study protocol for humans (Research Proposal 1405). Signed consent forms were obtained from participants enrolled in the study before questionnaires were administered and samples taken. Written informed consent was sought from parents of minors included in the study. The Seychelles Bureau of Standards gave the approval for the trapping and investigation of rats (A0157). All animal procedures carried out on rats were performed in accordance with the European Union legislation for the protection of animals used for scientific purposes (Directive 2010/63/EU). The research protocol’s ethical terms were defined under accreditation 03387 (FEDER POCT LeptOI 32913 research program) and were approved by the CYROI Institutional Animal Care and Use Committee (Comiteé d’Ethique du CYROI n° 114, IACUC certified by the French Ministry of Higher Education and Research). Veterinarians of the Veterinary Services of the Seychelles Agricultural Agency, Ministry of Agriculture and Fisheries, carried out sampling of dogs and cats.

### Inclusion of human acute cases

A national leptospirosis surveillance program was launched in Seychelles in December 2014. The study was designed as a prospective population-based survey conducted for one year from the 1^st^ December 2014 to the 30^th^ November 2015 on all acute febrile cases of unknown origin in Seychelles. Physicians were requested to refer all cases more than 13 years of age with acute fever of unknown origin and meeting the case definition (Box 1) to the reference leptospirosis clinic established at the Seychelles Hospital. Patients below 13 years were not included, in view of the larger spectrum of non-specific acute fever cases affecting this age group and the low prevalence of leptospirosis among this young age class based on local surveillance data. Referred patients were enrolled in the study after providing informed consent, and were assessed for severity and admitted to hospital if required as per established criteria. Demographic data *i*.*e*. age, sex and residential address were collected on all enrolled patients, as well as the outcome of clinical intervention, and a questionnaire was administered. Biological samples were collected to conduct an array of standard laboratory tests: 3 to 5 ml of whole blood was collected in tubes with and without anti-coagulant.

**Box 1. Case definitions and exclusion criteria**

**Case definitions**

***Suspected case***: Anybody aged 13 years and above reporting to a health center (private or governmental) during the 12-month period presenting with fever of ≥ 38°C for more than three days with or without any of the following signs and symptoms; headaches, myalgia, hemorrhagic manifestations in the absence of any definite diagnosis.

***Probable* case**: Anybody meeting the suspected case definition criteria with a Positive ELISA IgM as per the diagnostic criteria.

***Confirmed case***: Anybody meeting the suspected case definition criteria with a positive real-time PCR assay for pathogenic *Leptospira* spp. in blood and/or a positive MAT, a minimum four weeks after the onset of symptoms. A positive MAT was defined as one that displayed an infective Serogroup with a four-fold seroconversion in paired sera, or acute sera with a Serogroup displaying a minimum titer of 1:400. The infective Serogroup in sera that had coagglutinatinating titers had the serogroup displaying two titer orders more than the rest as the definitive infecting serogroup. For minors less than 18 years old, the accompanying guardian or parent was asked to give consent and to provide the relevant information.

**Exclusion criteria**

A person was excluded as a case if unwilling to participate in the interview and biological examinations and/or no samples were collected after enrolment.

### Animal sampling

Rats were trapped throughout Mahé at 12 sampling sites representing a spread over seven regions (Victoria, Victoria periphery, North, Centre, East, West and South); habitats were defined by descriptions of the sampling sites (S1 Table). Two trapping missions were conducted, one during the Southeast monsoonal season in June-July 2013, and the other in the Northwest monsoonal season in February-March 2014. All information including GPS coordinates and habitat types are provided as supplementary material (S1 Table). Trapping was conducted following a standard protocol [30] using wire cage traps baited with roasted coconuts. At each sampling site, 40 to 80 traps were placed 15 meters apart in line. Trapped animals were collected the following morning and brought back to a laboratory facility of the Ministry of Health. Animals were euthanized by cervical dislocation, blood collected by cardiac puncture, and dissected organs (kidney, liver, lung, spleen) were stored immediately in liquid nitrogen. Grinded fragments of fresh kidney tissue were immediately inoculated to culture medium (see below). *Rattus* species were identified using morphological characters [31] and animals identified as *R*. *rattus* were further sequenced at the cytochrome b (*cytb*) locus in order to avoid misdiagnosis within the *R*. *rattus* complex [32]. Dr. Jimmy Mélanie and Dr. Maria Tirant, respectively Principal Veterinary Officer and Veterinary Officer at the Seychelles Veterinary Section of the Seychelles Agricultural Agency in the Ministry of Environment and Agriculture, collected over a period of 4 months (December 2015 to March 2016) kidneys from healthy dogs and cats that were to be euthanized as part of the routine practice, *i*.*e*. from owners who wished to dispose of their unwanted animals. These kidney samples were stored in 70% ethanol at –80°C until shipment using dry ice.

### Nucleic acids preparation

Total nucleic acids were extracted from a pool of kidney, lung and spleen tissues collected from rats. Organs were dissected on ice, thin slices of approximately 20 mg of each tissue were pooled in 750 μL of DMEM (PAN-Biotech GmbH, Aidenbach, Germany) and grinded using a tissue lyser (QIAGEN, Les Ulis, France) and two 3 mm tungsten beads agitated at 25 Hz for 1 min. The resulting homogenate was centrifuged for 5 min at 10,000 rpm and 200 μL of the resulting supernatant was added to 200 μL of AVL buffer for subsequent extraction. In addition, 5 μL of bacteriophage MS2 (final concentration of 5μM) was added to one sample of every batch run and used as internal extraction control as previously described [33]. Extraction was performed on an EZ1 Advanced XL robot (QIAGEN, Les Ulis, France) using EZ1 virus extraction kit following the manufacturer’s instructions and using a final elution volume of 60 μL [18,19].

Nucleic acids were extracted from human serum samples either manually or using a QIAcube robot (QIAGEN, Les Ulis, France) as per manufacturer’s instructions using QIAGEN Viral minikit. A reverse transcription step was performed on 10 μL of the eluted total nucleic acids from human and animal origin using a GoScript reverse transcriptase (RT) kit (Promega, Charbonnières-les-Bains, France), by adding 1.25 μL of random primer hexamers, incubating at 80°C for 3 min and then holding at 4°C. To this mix was then added 4 μL of Buffer 5X, 2 μL of MgCl_2_ (25 mM), 1 μL of dNTPs (10 mM), 1 μL of RT (200 U/mL), 0.05 μL of RNAse inhibitor, and 0.7 μL of RNAse free H_2_O. Reverse transcriptions were carried out using the following conditions: 25°C for 5 min, 42°C for 60 min, 70°C for 15 min and then held at 17°C.

As dog and cat kidneys were preserved in 70% ethanol, samples were first rehydrated overnight in osmosis water before carrying out DNA extraction using DNeasy Blood and Tissue extraction kit (QIAGEN) following manufacturer’s instructions. Reverse transcriptions were also carried out on these samples following the conditions mentioned above. All produced cDNAs were stored at –80°C until PCR detection.

### PCR detection and genotyping through MLST

The screening of cDNA using a probe-based real-time PCR (Polymerase Chain Reaction) for pathogenic *Leptospira* spp. was done adapting Smythe’s protocol to target the *rrs* (16S) gene [34]. Amplifications were performed in 25 μL containing 12.5 μL of Absolute Blue real-time PCR Low Rox Mix (Thermo Scientific, Waltham, MA, USA), 0.5 μL (10 μM) (initial stock concentrations shown) of each primer, 0.4 μL (10 μM) probe and 6.1 μL of nuclease free water. The PCR conditions were 95°C for 15 min, followed by 45 cycles at 94°C for 30 sec and 60°C for 1 min. For human samples detection, a cut-off criterion was set at Cycle threshold (Ct) <35 for positivity following Ahmed *et al*. [35]. *Leptospira interrogans* DNA serially diluted and leading to a Ct of 30 was used as a positive control for RT and end-point PCRs. A minimum of three distinct negative (water) controls were performed for each RT-PCR run and one single negative (water) control for each end-point PCR. PCR detection was first performed in triplicates and samples producing at least two positive reactions were considered positive, while samples producing a single positive reaction were submitted to an additional triplicated PCR. Moreover, samples with amplification at only one or two out of six real-time PCR runs were not considered positive unless a *Leptospira* sequence was achieved. Additionally, samples that had 35<Ct<40 for more than two replicates out of six were considered positive. Animal samples were submitted to a single real-time PCR with a cut off criterion set at Ct<45. Genotyping of positive samples was carried out through Multi Locus Sequence Typing (MLST) scheme#3 (http://pubmlst.org [36]). This scheme was chosen instead of two other available schemes as a number of investigations on other islands of the SWIO region have been carried out using this same MLST scheme. The amplification of *adk*, *icd*A, *lipL*32, *lipL*41, *rrs*2 and *secY* genes was performed using generic primers [36]. In case of PCR failure, samples were submitted to an alternative PCR using degenerated primers [18] and/or to an alternative amplification of *rrs*2 gene using previously published LA/LB primers [37]. All amplicons were sequenced on both strands (GenoScreen, Lille, France) and sequences were edited using Geneious 9.1.3 [38]. Original sequence types (STs) were deposited on the pubMLST database. DNA sequences were deposited on GenBank and accession numbers are listed on S2 Table.

### *Leptospira* culture from rat kidneys

Kidneys from freshly dissected rats were aseptically sectioned and finely minced with a blade before inoculating three distinct media: (i) Ellinghausen-McCullough-Johnson-Harris (EMJH) liquid basal medium (Difco, Detroit, MI, USA) supplemented with Albumin Fatty Acid Supplement (AFAS, purchased at OIE and National Collaborating Centre for Reference and Research on Leptospirosis Academic Medical Center, Department of Medical Microbiology, Amsterdam) [39,40]; (ii) EMJH liquid basal medium supplemented with AFAS, rabbit serum and foetal calf serum (1% each); and (iii) semisolid Fletcher medium (Difco, Detroit, MI, USA) supplemented with rabbit serum (8%). All media were supplemented with 5-fluorouracil (5-FU) at a final concentration of 200 μg.mL^-1^. Cultures were incubated at 28°C, visually checked for the presence of *Leptospira* using a dark field microscope once a week for four months, and positive cultures were further sub-cultured in fresh EMJH liquid basal medium supplemented with AFAS but deprived of 5-FU. A detailed protocol is available at http://dx.doi.org/10.17504/protocols.io.ifccbiw.

### Serological screening through ELISA and Microscopic Agglutination Test (MAT)

All acute human sera were screened through an in-house IgM ELISA test using 96-well Immulon 1B polystyrene plates coated with *Leptospira biflexa* serogroup Patoc antigen (already prepared at 11×10^8^ leptospires/mL from cultures and stored at 4°C). The antigen preparation was used at a dilution of 1:30 to test all 223 human sera on ETI-Max 3000 (DiaSorin, Saluggia, Italy) at the GHSR-CHU (Groupe Hospitalier Sud Réunion-Centre Hospitalier Universitaire) hospital of Saint Pierre in Reunion Island. Absorbances were read at 450/620 nm. MAT was based on a panel of twenty *Leptospira* strains (see S3 Table) allowing detecting most serogroups that have been previously reported in humans [13,14,25,41] and animals [42] in the SWIO islands. All patients enrolled in the prospective study were tested by MAT using the initial blood sample (acute phase) to measure the prevalence of antibodies to *Leptospira* in the cohort (reflecting either ongoing, recent or old infections). For 46 patients of the cohort, we could obtain a second blood sample at least four weeks after the onset of the first signs and symptoms (convalescent phase) and these 46 paired sera were tittered with MAT. Sera were tested at dilutions ranging from 1:50 to 1:3200. A MAT titer of more than or equal to 1:100 (cut off value of the test) indicated a seropositive sample and the reactive serogroup as the one allowing agglutination at two titer orders more than the other coagglutinins. MAT serology was diagnostic of acute leptospirosis only if the MAT titer was ≥ 1:400 on the acute phase sample and/or demonstrated a four-fold increase in titer (*i*.*e*. seroconversion) on paired sera. Since Icterohaemorrhagiae was the serogroup previously reported as most prevalent in human acute cases [25] and as Patoc cross-reacts with most serogroups, we carried out MAT in two steps. First, a screen at 1:100 using Patoc and Icterohaemorrhagiae serogroups allowed highlighting putative positive samples. Second, all the samples reactive against Patoc and/or Icterohaemorrhagiae were serially diluted, up to a titer of 1:3200 and submitted to MAT using the full panel listed in S3 Table.

### Diagnostic criteria of human leptospirosis

Overall, a patient was considered positive for leptospirosis if one of the following conditions was fulfilled: (i) MAT titer of acute serum ≥1:400; (ii) evidence of seroconversion on paired sera attested by a fourfold increase of MAT titers; (iii) real-time PCR with Ct<35 for at least two replicates out of six; (iv) real-time PCR with 35<Ct<40 for at least 3 replicates out of six; (v) real-time PCR with Ct<40 on one or two attempts and simultaneously positive for IgM by ELISA; (vi) real-time PCR with 35<Ct<40 and with at least a sequence achieved on one of MLST loci. Samples that were positive for IgM ELISA only were considered negative due to the possibility of rheumatoid factors giving a false positivity [43] and also for the well-known long-term persistence of anti-*Leptospira* IgM antibodies months and years after acute infection [44,45].

### Mapping

Georeferencing and mapping of human cases and *Leptospira* positive *Rattus* spp. was carried out using QGIS v2.18.0 “Las Palmas”, [46] freely available at http://www.qgis.org/en/site/.

### Statistical analyses

We investigated the effects of different variables on the infection status of rats: host “Species” (*R*. *norvegicus vs R*. *rattus*) and “Maturity” (juvenile *vs* adult), “Seasons” (wet *vs* dry), as well as environmentally variables such as “UrbanOrRural” (representing the type of habitat, urban or rural), and “Region” (Victoria, Victoria periphery, North, Centre, East, West and South). Variable “UrbanOrRural” was determined in accordance to descriptions recorded during sampling at each site (refer S1 Table): “urban” habitats are built or heavily disturbed habitats while “rural” sites are residential, mixed-agricultural or natural habitats. Pairs of variables were compared using Fisher’s exact test for count data. Generalized linear models including “Species”, “Maturity”, “Seasons”, “UrbanOrRural”, and “Region” as explicative variables were performed using a binomial distribution and logit link function (log-likelihood type 1 test). Analyses were conducted using “R” software [47].

### Minimum-spanning tree

A minimum-spanning tree (MST) was built using goeBURST Full MST algorithm (PHYLOViZ 1.1, 2014), by concatenating six MLST gene markers (*adk*, *icd*A, *lipL*32, *lipL*41, *rrs*2 and *secY*; fused in that order) and comparing with previously published STs found in various hosts worldwide.

## Results

### Incidence of human leptospirosis in Seychelles

Overall, 225 patients out of 226 presenting with acute fever of unknown origin were enrolled in the study (one patient refused to participate). Two patients for whom no sample could be obtained were excluded, leaving a total of 223 patients effectively investigated for acute leptospirosis. There were 23 females and 198 males (gender information was missing for two patients) with a mean age of 33 years old (range 13 years– 60 years) and a median of 34 years old. A total of 51 patients (49 males and 2 females) were diagnosed with leptospirosis (see below) of whom 6 patients (5 males and 1 female) died of their disease, leading to a case fatality rate for leptospirosis of 11.8%.

**Fig 1:**
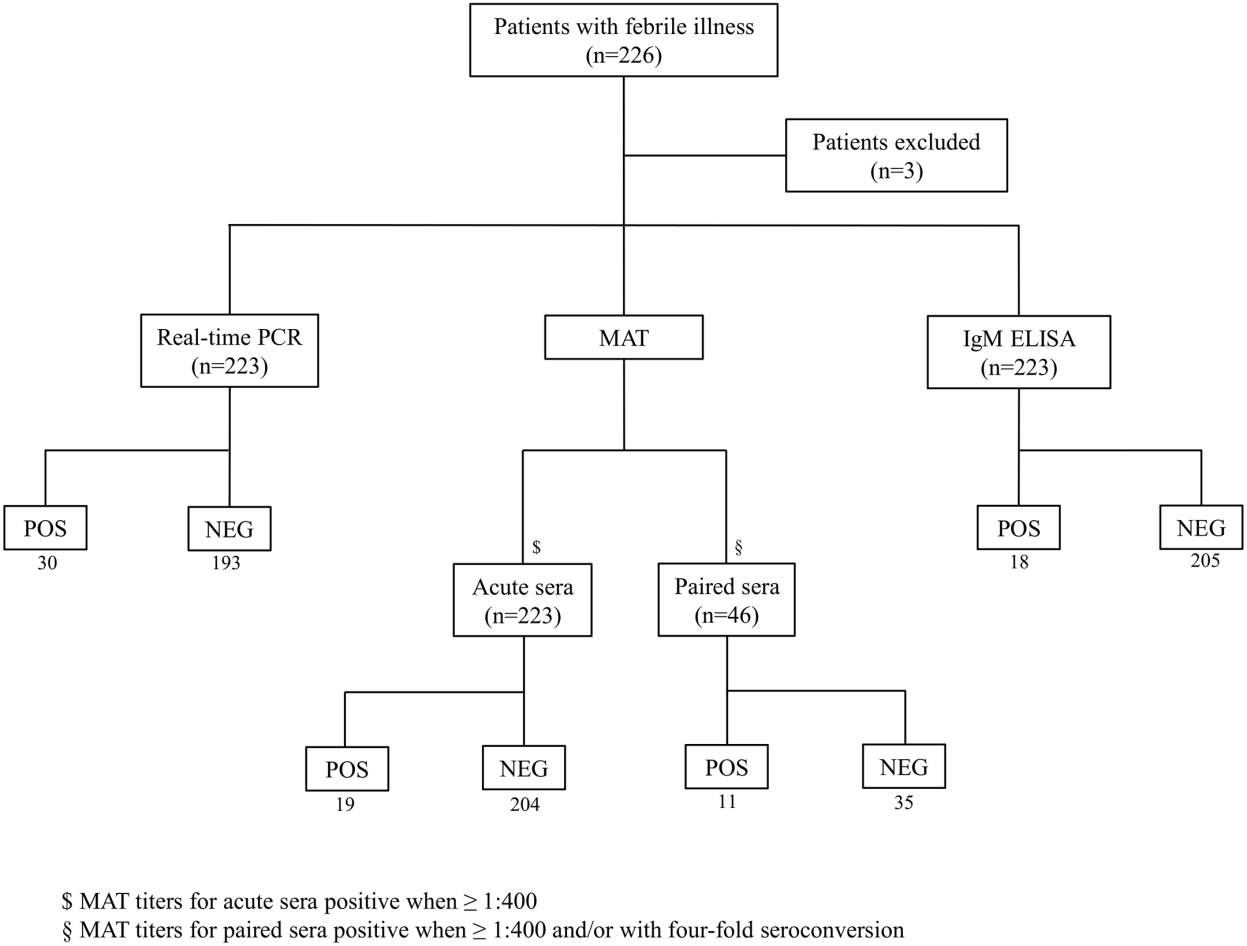
Diagnostic flow chart of tests done, number of enrolled patients and diagnostic results.

There was a moderate agreement (44.5%) between MAT and real-time PCR results: out of 46-paired sera (representing 20.6% of all patients), 11 were MAT positive out of which six were also real-time PCR positive. Of the 35 paired samples that were MAT negative, 88.6% were also negative by real-time PCR. Agreement by Cohen’s Kappa (Table 1) between confirmed positives as defined by the diagnostic criteria and the different performed tests were categorized as: good for PCR (87.8%) and MAT (81.4%), and moderate for IgM by ELISA (45.7%). A flow chart of the tests conducted and the positive and negative results are shown in Fig 1.

**Table 1:** PCR, MAT and IgM ELISA, agreement and test performance values. Number of positive and negative samples is defined as per the diagnostic criteria. Kappa (κ) / [Agreement Criteria]: ≤ 20 / [Poor]; 21–40 / [Fair]; 41–60 / [Moderate]; 61–80 / [Substantial] and 81–100 / [Good].

		Tests		
		PCR	MAT	IgM ELISA
Confirmed positive (n = 51)	Positive	30	19	18
	Negative	21	32	33
Confirmed negative (n = 172)	Positive	0	0	0
	Negative	172	172	172
Sensitivity (%)	Value	58.8	37.3	35.3
	95% CI	44.2–72.4	24.1–51.9	22.4–49.9
Specificity (%)	Value	100	100	100
	95% CI	97.9–100	97.9–100	97.9–100
Positive Predictive Value	Value	100	100	100
	95% CI	97.9–100	97.9–100	97.9–100
Negative Predictive Value	Value	89.1	84.3	83.9
	95% CI	85.5–91.9	81.3–86.9	81.0–86.5
Cohen's Kappa (%)	Value	87.8	81.4	45.7
	95% CI	79.7–93.5	72.4–88.5	35.7–56.0

**Fig 2:**
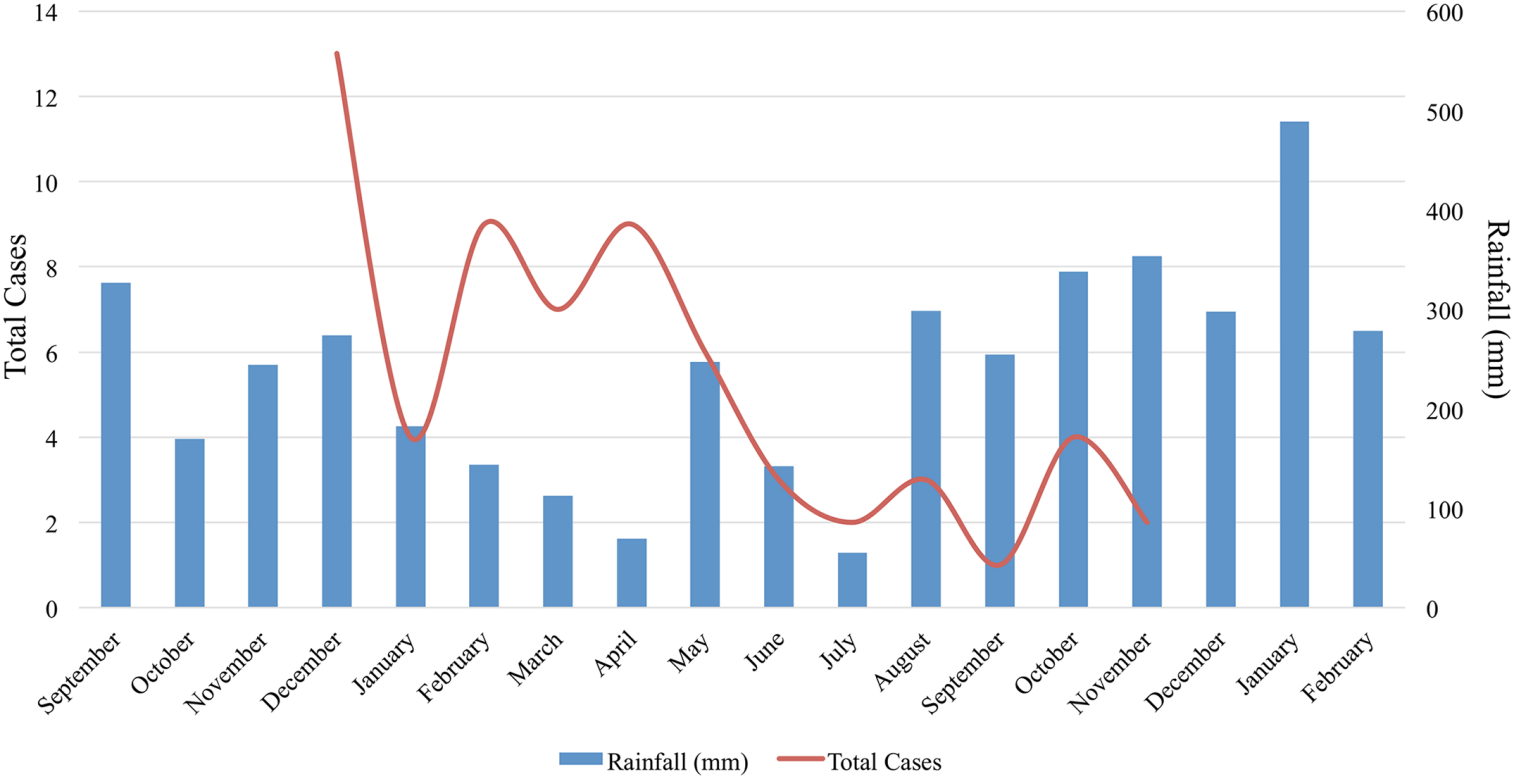
Leptospirosis total positive cases over a one-year period (1^st^ December 2014 to 30^th^ November 2015) in relation to rainfall data (mm) obtained from Seychelles airport and including three months before and after the study period.

Altogether, 51 patients fulfilled the diagnostic criteria of acute leptospirosis either by real-time PCR (n = 30) (as per criteria (iii) and (iv)) IgM ELISA with PCR (n = 9) (as per criteria (v)) and confirmed MAT (n = 19) (as per criteria (i) and (ii)), as well as the aforementioned sample for which a sequence was produced (diagnostic criteria (vi)). Considering the current population of Seychelles [23], incidence of human leptospirosis was evaluated to be 54.6 (95% CI 40.7–71.8) per 100,000. Temporal analysis of the prevalence of cases over the one-year period from December 2014 to December 2015 shows a decreasing trend from the January to March period, corresponding to the humid Northwest monsoon, towards the usually dry season, where fewer cases were reported (Fig 2). There is some seasonality depicted by a high number of cases after a period of high rainfall, although not as sharp as that reported in other insular ecosystems of the region (see Discussion).

In order to identify the most prevalent serogroups, we carried out MAT screening of all patients enrolled in the study using the blood sample collected at inclusion (acute phase serum samples). Forty-five acute phase sera out of 223 tested seropositive by MAT (*i*.*e*. titer > 100) at inclusion. Among the seropositives, the serogroup Icterohaemorrhagiae was dominant (n = 8), followed by Autumnalis (n = 5), Hurstbridge (n = 4), Australis (n = 4), Djasiman (n = 3) and Sejroe (n = 1). The serogroups Ballum and Canicola, previously reported in Seychelles [25], were not detected in our sample while the previously identified serogroup Louisiana was not included in our panel. We did have two sera that were Patoc positive but did not agglutinate with any of the 20 reference strains of our panel. As a significantly large number (n = 18) of sera in our sample set displayed cross-agglutination, we identified the infective serogroup as the one allowing agglutination at two titer orders more than the other coagglutinins. According to this criterion, the distribution of major cross-agglutinating serogroups, in decreasing order, were Icterohaemorrhagiae (n = 18), Autumnalis (n = 8) and Hurstbridge (n = 8). A single sample displayed equal cross-agglutination to Pomona and Hardjobovis serogroups (see S4 Table for tabulated MAT results).

### Genetic diversity of *Leptospira* infecting humans

MLST sequences were produced for 24 out of the 32 real-time PCR positive patients, distributed for the six gene loci as follows: *adk* (n = 21), *icd*A (n = 19), *lipL*32 (n = 22), *lipL*41 (n = 21), *rrs*2 (n = 22) and *secY* (n = 20). *Leptospira* sequences that were obtained from these 24 patients were all identified as *Leptospira interrogans*. Complete six-loci MLST was achieved for 18 patients leading to three different STs: one was identified in pubMLST database as ST02, whereas two STs were not previously reported/registered in the database and thus considered as novel. These two STs were submitted to the pubMLST database and consequently assigned as ST142 and ST143. Thus ST02 (n = 4), ST142 (n = 11) and ST143 (n = 3) represented 22.2%, 61.1% and 16.7% of positive human samples with full MLST, respectively. In order to use the whole sequence data, we arbitrarily assigned an ST to those samples for which only partial genotyping was achieved. For this, after establishing that all alleles or combination of alleles were compatible with ST02, ST142 or ST143, we included in the analysis those human samples for which the obtained sequences allowed unambiguous ST assignation. This allowed us to assign an ST to 21 out of the 24 fully or partially sequenced human samples. With this dataset, ST02 (n = 7), ST142 (n = 11) and ST143 (n = 3) were found in 33.3%, 52.4% and 14.3% of human samples, respectively.

### *Leptospira* carriage and genetic diversity in rats

**Fig 3:**
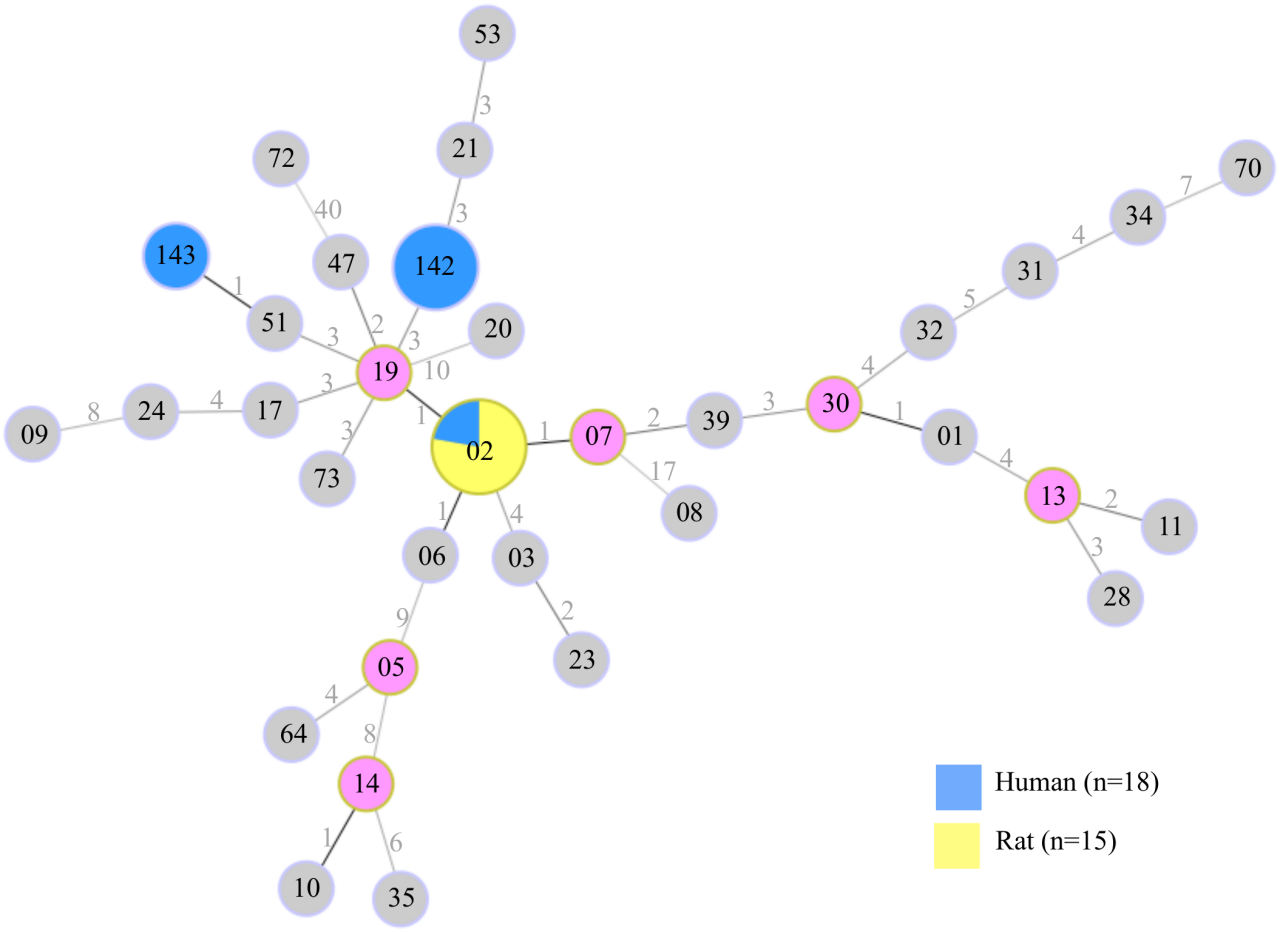
Minimum-spanning tree of *Leptospira interrogans* sequence types (STs) based on the MLST scheme #3 (http://www.pubmlst.org/leptospira/). Sequence Types from humans (in blue) and rats (in yellow) from Seychelles were included into a network constructed with previously published STs reported from various hosts worldwide and shown in grey circles. Group founders are shown in purple circles. The circle sizes of ST02, ST142 and ST143 reflect the relative abundance of each ST in the data set acquired from human and rat samples. The numbers indicated on branches represent the number of mutations between each ST.

Altogether 739 rats were sampled and screened for *Leptospira* carriage, leading to an overall prevalence of 7.7%. Genotyping provided sequences for 34 out of the 57 positive animals (see S1 Table) distributed as follows: 24 sequences for *adk* gene, 18 sequences for *icd*A, 21 sequences for *lipL*32, 20 sequences for *lipL*41, 29 sequences for *rrs*2 and 27 sequences for *secY*. Additionally, 13 sequences were achieved for *rrs*2 gene using LA/LB primers [37] revealing sequences for two additional samples which were not successfully genotyped using MLST scheme#3 [36]. Full MLST was obtained from all 12 *Leptospira* positive cultures attempted from 74 rat fresh kidney tissues as well as from three uncultured tissue samples. Full genotyping of these 15 samples revealed the exclusive presence of *L*. *interrogans* ST02. Following the same procedure as that used for human samples analyses, we arbitrarily assigned an ST to those samples for which only partial genotyping was achieved. The allelic profiles of these remaining samples were all compatible with ST02, ST142 or ST143 but only nine allowed unambiguous ST assignation and were all indicative of ST02. A minimum-spanning tree presented in Fig 3 shows alleles differences between all three STs in fully genotyped human and rat samples.

### Influence of biotic/abiotic variables on infection prevalence in rats

**Fig 4:**
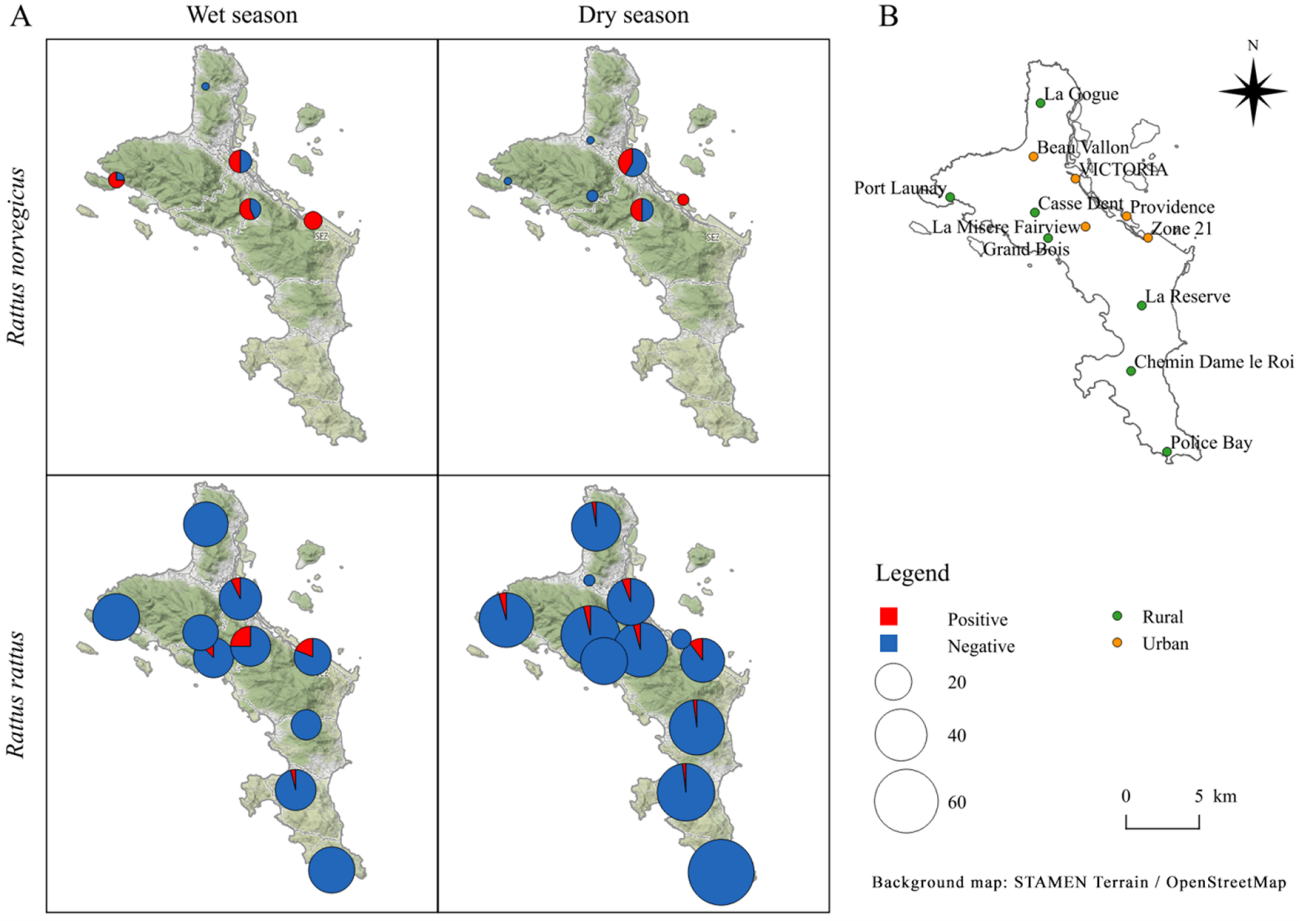
Distribution of sampled rats on Mahé Island, with infection status. **A**. The quadrants show the distribution of *Leptospira*-infected *Rattus norvegicus* and *R*. *rattus* plotted by Wet Season (Northwest monsoon, February-March 2014, n = 464) and Dry Season (Southeast monsoon, June-July 2013, n = 275). Circle sizes represent the relative number of rats captured at each site with a representation of the positives (in red) and negatives (in blue). **B**. Sampling sites are plotted with urban and rural habitats appearing in orange and green, respectively (see S1 Table for details including GPS coordinates). Maps were produced using QGIS, and the Mahé Island shape file obtained from OpenStreetMap (https://www.openstreetmap.org).

Overall, *Leptospira* prevalence was significantly higher (p-value < 0.0001) in *R*. *norvegicus* (52.9%, n = 51) than in *R*. *rattus* (4.4%, n = 688). Several biotic and abiotic variables affected the prevalence of *Leptospira* renal carriage in rats, we describe hereafter the influence of each analysed variable. Infection prevalence appeared significantly affected by the sampling season. Prevalence of *Leptospira* carriage was 5.4% (n = 464) during the dry season *vs*. 11.6% (n = 275) during the wet season (Fig 4) (p-value = 0.003). An analysis of urban versus rural habitats irrespective of rat species and season showed that there was also a significantly higher positivity rate in urban (18.7%; n = 230) than in rural (2.8%; n = 509) habitats (p-value < 0.0001; see Fig 4). When each rat species was analysed separately, the difference in infection prevalence was not significant for *R*. *norvegicus* (55.8% in urban *vs*. 37.5% in rural) but remained significant for *R*. *rattus* (10.2% in urban *vs*. 2.2% in rural, p-value < 0.0001). The higher prevalence in urban habitat was still significant when each season was analyzed independently: *Leptospira* carriage in rats was 13% (n = 138) in urban and 2.2% (n = 326) in rural habitats during the dry season (p-value <0.0001) while during the humid season, infection reached 27% (n = 92) and 3.8% (n = 183) in urban and rural habitats (p-value = 0.009), respectively.

When infection prevalence was compared for each season independently, it appeared that *R*. *norvegicus* was still significantly more infected than *R*. *rattus* during both wet (64% *vs* 6.4%; p-value < 0.0001) and dry seasons (42.3% *vs* 3.2%; p-value < 0.0001). *Leptospira* carriage was also significantly higher in *R*. *norvegicus* than in *R*. *rattus* whether animals were trapped in urban (55.8% *vs* 10.2%; p-value < 0.0001) or rural habitats (37.5% *vs* 2.2%; p-value < 0.0001).

Overall, 4.5% (n = 5) of juvenile rats (n = 110) were carriers of *Leptospira*, representing 0.7% of the total rats sampled. Conversely 8.3% of adult rats (n = 629) were carriers of *Leptospira* representing 7% of total rats sampled. An analysis of maturity status of *Rattus* species in relation to *Leptospira* carriage showed that adult *R*. *norvegicus* were more infected (56.3%, n = 48) than adult *R*. *rattus* (4.3%, n = 581; p-value < 0.0001); whereas there was no significant difference detected amongst juveniles, possibly due to the very low number of caught *R*. *norvegicus* juveniles (n = 3). Both adult (p-value < 0.0001) and juvenile rats (p-value = 0.005) were more infected in the urban than in the rural habitats, adult rats being more infected in the wet (11.7%, n = 248) than in the dry (6.0%, n = 381) season (p-value = 0.017).

We tested the distribution of *Rattus* spp. in urban *vs*. rural habitats, showing that both rat species were unevenly distributed (see Fig 4), *R*. *norvegicus* colonizing preferentially urbanized habitats (84.3% of sampled rats, n = 51) while *R*. *rattus* was dominant in rural settings (72.8% of sampled rats, n = 688; p-value < 0.0001). Hence, the observed significant distribution of infected rats may be indirectly related to the uneven spatial distribution of *Rattus* spp. We addressed this hypothesis by performing a generalized linear model (glm) including all variables (*i*.*e*. “Species”, “Seasons”, “UrbanOrRural”, “Maturity” and “Region”) acting alone or in interaction. Using this model, the effect of *Leptospira* carriage amongst rats showed a borderline effect of season (Wet > Dry; p-value < 0.1), whereas increasingly significant differences of *Leptospira* carriage were observed for habitat (urban > rural; p-value < 0.05) and *Rattus* species (*R*. *norvegicus* > *R*. *rattus*; p-value < 0.001). Performing a glm with “Species” and “Region” as explicative variables highlighted significant *Leptospira* infection in *R*. *norvegicus* (p < 0.0001) in the East region (p < 0.005) of Mahé. No other effect was highlighted for “Maturity” and “Region” variables. Further analyses of the interactions of variables “Species”, “Seasons”, “UrbanOrRural”, “Region” and “Maturity” did not highlight any significant interaction between these variables when taken together. A stratified general linear model analysis based on host species (*i*.*e*. by *R*. *norvegicus* and *R*. *rattus* separately) against all the other variables did not reveal any significant interaction between variables either.

### *Leptospira* prevalence and diversity in dogs and cats

Following the investigation of rats and humans, it appeared that rats were likely not the only reservoir involved in human leptospirosis, as two thirds of PCR confirmed human cases were infected with a *L*. *interrogans* ST that was virtually absent from our genotyped rats sample. In an attempt to explore other possible reservoirs, we collected kidney samples from 12 cats and 24 dogs. One cat (C13; Ct = 40) and one dog (D18; Ct = 39) were diagnosed as infected with *Leptospira* by real-time PCR. Only the dog sample allowed the production of sequences for *adk*, *lipL*32 and *lipL*41. Although no complete MLST could be achieved, the allelic profile obtained using the three sequenced *loci* was consistent with the novel ST142, representing the most common ST found in human leptospirosis cases.

## Discussion

We report a human incidence of 54.6 (95% CI 40.7–71.8) per 100,000 inhabitants in Seychelles. This incidence is higher than that reported in Reunion island (8.2 cases per 100,000) and second in the region following Mayotte island, which displays a comparable high incidence (74.5 cases per 100,000) [12]. This incidence falls within the mean incidence of 41.1 (95% CI 29.5–55.7), calculated from a ten-year period (2005–2014) based on routine data collated by the Health Statistics Unit (Epidemiology and Statistics Section, Public Health Authority). However, the differences in incidence to previous studies in spite of the overlapping confidence intervals, from 101 cases per 100,000 previously reported in 1995–96 [25], to 54.6 cases per 100,000 in 2014–15 reported herein, requires some discussion. It must be emphasized that methods implemented in both studies are close but not identical. We carried out real-time PCR, IgM ELISA and MAT, while the previous study used end-point PCR and MAT only as the defined diagnostic criteria. The real-time PCR used herein is highly sensitive, likely because of the short stretch of amplified DNA. Considering our diagnostic criteria, we state that our screening was at least as sensitive as that implemented 20 years ago. However, the case definition used in this study was more restricted than the previous study and may have missed early cases presenting without fever or late stage cases presenting with jaundice. Lastly, for feasibility reasons, all human samples were first screened through MAT using Icterohaemorrhagiae and Patoc serovars only, which might have led to some false negative samples. Hence, the incidence reported herein may actually be underestimated.

Although lower than previously reported [25], the incidence of human leptospirosis in Seychelles remains high, indicating that Seychelles still has a heavy disease burden. The collected demographic data reveals a strong bias towards males in our sample (90.5% males vs. 9.5% females). This bias is unexpected as all patients with acute febrile illnesses were included in the study, but is actually close to biases reported in two previously published leptospirosis studies in Seychelles (89% and 84% males reported in [24] and [25], respectively). Part of the explanation is that leptospirosis is actually an important portion of acute fever illnesses and is well known to be much higher in males than females [48], but such a high bias is still difficult to understand with our limited knowledge of acute febrile illnesses in Seychelles. We cannot rule out that although the protocol was designed to include all patients presenting with acute febrile symptoms of unknown origin, physicians who were informed of the general objectives of the project, *i*.*e*. an estimation of leptospirosis burden, may have spontaneously biased the recruitment towards putative leptospirosis patients, and hence towards males known to be significantly more affected than females by the disease. If so, reported figures may actually be under estimated. Interestingly, our data confirm the high case fatality rate of leptospirosis in Seychelles and the maintenance of the severity of the disease over the years: 11.8% in 2014–2015 versus 16% in 1988–1990 [24] to 8% in 1995–1996 [25]. Despite considerable improvements in the diagnosis over the last 20 to 30 years, the morbidity and severity of the disease does not seem to have decreased. The MAT data are overall in accordance with the major serogroups previously reported by Yersin *et al*. [25]. The high amount of cross-agglutinations that we report may be an indirect indicator of how much the Seychellois population is exposed to *Leptospira* with the presence of co-agglutinins possibly indicative of intense exposure and iterative reinfections.

Molecular investigation of rats and human acute cases brings in original data that enlightens the epidemiology of the disease in Seychelles. We report a very limited *Leptospira* diversity within rats and human cases. Indeed, *L*. *interrogans* was the single species found in both human acute cases and rats. When overlaying leptospiral diversity found in rats and human acute cases, it appears that ST02 is the only ST detected in rats while it is detected in only 22.2% (n = 18) of clinical samples with full MLST profile, and in 33.3% of clinical samples with full and partial MLST profile (n = 24). Noteworthily, two novel STs reported herein, namely ST142 and ST143, are dominant in human acute cases but were not detected in any of the fully or partially genotyped rats. Although it cannot be excluded that our sampling was not sufficient to capture the whole diversity of *Leptospira* maintained by rats, we can reasonably propose that other animal reservoir(s) or carrier(s) are actually involved in the epidemiology of human leptospirosis. Indeed, prevalence of *Leptospira* carriage reported herein in rats (7.7%, n = 739) is notably lower than that reported in other islands of the region such as Reunion (36.3%, n = 732) or Mayotte Islands (15.9%, n = 289) [49,50]. A recent report has suggested that domestic animals such as dogs may act as possible vectors in the transmission of *Leptospira* in the environment and consequently indirectly affecting humans [66]. Although serogroup Canicola commonly associated with dogs was not detected in our samples except in co-agglutination, this important issue needs to be addressed considering the abundance of stray dogs in the islands of Seychelles. Interestingly, the probable presence of ST142 in one dog (substantiated by sequences at three MLST loci), suggests that dogs may actually be shedders of this ST, found in over 50% of genotyped human samples. The situation in Seychelles can be compared to that occurring in Reunion Island where rats were found exclusively reservoirs of ST02 as well. In this French Island, two *Leptospira interrogans* lineages were found in humans, ST02 and ST34, and partial sequencing of *Leptospira* supports the presence of both lineages in dogs [49]. Although the present sampling setup was not designed to investigate the dogs’ compartment, our results strongly call for a proper exploration of these as well as other domesticated animals or wild fauna.

Our study addresses the role of biotic and abiotic factors in the epidemiology of *Leptospira* carriage in rats, which can be overlaid on the dynamics of human leptospirosis. The data shows that there is a higher prevalence of *Leptospira* carriage in rats during the humid season than in the dry season, which has similarly been described in other insular tropical territories like Martinique and Reunion Islands [51,52]. This higher prevalence can be reasonably attributed to humid conditions prone to the maintenance of *L*. *interrogans* in the environment for long periods [53–56]. However, leptospirosis cases can be observed throughout the year, which can be best explained by an elevated and constant temperature all year round together with bouts of rainfall occurring even during the traditionally dry period (June-August). Altogether, it appears that the seasonality of leptospirosis in Seychelles is not as sharp as that reported in Mayotte and Reunion islands [11,57], likely because of a more equatorial rather than tropical climate in Seychelles and a more pronounced rainfall seasonality found in the two French overseas territories. However, rainfall might not explain all the variability observed, and long-term surveillance may help pinpoint other variables at play.

The importance of urbanization in predicting *Leptospira* carriage in rats is significant (Fig 4). In fact, 68% of *Leptospira*-positive rats were sampled in the urban environment during the dry season and this increased to 78% during the wet season. A general trend highlighted by our data is that habitat degradation is positively correlated with *Leptospira* carriage in rats. For instance, the only site with no infection detected in rats during both sampling seasons was at the southernmost extremity of Mahé at Police Bay, which is actually a natural habitat. Other natural environments such as La Réserve, Casse-dent, La Gogue and Grand Bois, sheltered infected rats but with lower prevalence as compared to those trapped in urbanized sites. Therefore, the traditional association of leptospirosis with occupational and environmental risk factors such as farming and agricultural zones, although still of importance in many countries, may not be so much an issue compared to the exposure of human populations to urban environments as shown in the case of Seychelles. It has to be noted that few residences are located in what we have classified as urban environments of Victoria, as these contain mainly office blocks, banks, port, factories, and commercial zones. People come to work or for leisure activities in these zones however mostly reside in other areas, hence human exposure in this zone may be low although the infection in the urban zones could play a role as a reservoir of the pathogen, which could diffuse to contiguous areas or to other animal species.

A glowing pattern highlighted by our data is that *R*. *norvegicus* appears dramatically more infected than *R*. *rattus*. When urban and rural environments were analyzed independently, *R*. *norvegicus* was again significantly more infected than its sister species. Hence, it appears that in Seychelles, *R*. *norvegicus* might be epidemiologically much more involved than *R*. *rattus* in the contamination of the environment with pathogenic *Leptospira*. However, the abundance of each *Rattus* species in each habitat should be comprehensively addressed in order to conclude on the weight of each respective species in the epidemiology of human disease. The distribution of *R*. *norvegicus* on Mahé must be overlaid upon ecological studies and observations done in Seychelles [28,58,59]. The distribution of *R*. *norvegicus* in the mainly urban areas on Mahé is consistent with studies showing its commensalism to humans [60,61]. However, such urban distribution contrasts with its past distribution (before the species was eradicated) on smaller islands of Seychelles (*i*.*e*. Frégate, D’Arros and Conception) where *R*. *rattus* was absent [62] and with reports from other countries where *R*. *norvegicus* is distributed in all habitats including rural areas [63].

Observer ecological records place the introduction of *R*. *norvegicus* in Seychelles relatively recently (*i*.*e*. within the last century), rats being first reported from D’Arros and Conception islands (later found to be occupied solely by *R*. *norvegicus*) in 1944 and 1965 respectively [28], whereas by comparison, *R*. *rattus* was first reported in Seychelles in 1773 and was probably previously present on the islands [28,58]. The current distribution of both *Rattus* spp., and particularly the more restricted distribution of *R*. *norvegicus* on Mahé, probably results from the more recent colonization of the latter, its stronger human synantropism and preference for wetter habitats (which in turn also correlates with higher *Leptospira* carriage in rats), but the respective importance of these factors is still to be investigated.

In addition to the present investigation of animals, a recent study has notified the presence of a *L*. *kirschneri*-like species (sequenced on one gene only) in *Pteropus seychellensis* (Dietrich *et al*, submitted), a common frugivorous bat in Seychelles. The absence of this bacterial species in our human samples infers a lack of any role of these bats in the epidemiology of the disease. Again, the situation in Seychelles is quite similar to that recently reported on Reunion Island where an insectivorous endemic bat species, *Mormopterus francoismoutoui*, massively excretes *L*. *borgpetersenii* although this specific bat-borne lineage was not found in human cases or in rats [49]. Also, our study did not investigate a third and more discrete species of rodent present in Seychelles, the House mouse *Mus musculus*, a highly commensal species identified as a *Leptospira* carrier in other island countries. Lastly, studies around the world have revealed the existence of *Leptospira* carriers in animals other than mammals, including birds and reptiles [64], and even invertebrates [65], stimulating further exploration of alternative animal carriers in Seychelles.

### Conclusion

From a biogeographic perspective, the present study not only completes previous studies carried out in Seychelles but also brings in original data showing that the South Western Indian Ocean islands actually shelter distinct epidemiological situations. Some oceanic islands such as Seychelles and Reunion Island [49] host a narrow diversity of cosmopolitan pathogenic *Leptospira* possibly of recent introduction, while other territories such as Mayotte [13,14] and Madagascar [17,18,19] are host to a much higher *Leptospira* diversity, including endemic lineages of medical importance [50]. Altogether, the presented data further confirms that insular ecosystems facilitate the exploration of infectious diseases, as these environmental settings are home to peculiar species assemblages that are in turn involved in unique transmission pathways.

This study has completed previously sparse information regarding human leptospirosis in Seychelles, which can still be considered as one of the countries with highest incidence worldwide. Presented results may guide the public health intervention strategies in the prevention of human leptospirosis and the control of animal reservoirs in Seychelles. The patterns of environmental exposure revealed herein support rat control efforts targeting the urban areas of Seychelles as they are expected to have a significant impact in reducing the risk of leptospirosis transmission and hence reducing the overall incidence in humans. However, genotyping of *Leptospira* from animals and human acute cases reveals that rats are potentially involved in less than a third of human infections. The insignificant change in mortality caused by leptospirosis together with a persistent high incidence of the disease in humans, highlights undetectable improvement in management of acute cases in humans worsened by a limited efficiency of preventive measures. This may result from insufficient rodent control measures or, as suggested by our study, from a misidentification of the main reservoir(s) still to be identified and controlled.

## Supporting information

S1 Table *Rattus* samples tested by *Leptospira* 16S real-time PCR and/or culture, including MLST typing data, habitat type, season, sexual maturity and GPS coordinates. (XLSX)

S2 Table GenBank accession numbers of sequences generated in this study that are representative of the *Leptospira interrogans* STs found. (XLSX)

S3 Table List of *Leptospira* spp. strains used for the MAT panel. (DOCX)

S4 Table Table of results for ELISA IgM, PCR consolidated (after two triplicate runs), MAT serogrouping (on acute and paired human sera) and sequence type of fully genotyped samples. (XLSX)

S1 Checklist STROBE checklist. (DOC)
