# Supplementary material for: Human leptospirosis in Seychelles: A prospective study confirms the heavy burden of the disease but suggests that rats are not the main reservoir
Source: PLoS Negl Trop Dis. 2017 Aug 28;11(8):e0005831. doi: 10.1371/journal.pntd.0005831 (PMC5591009; doi:10.1371/journal.pntd.0005831)
Supplement: S3 Table — (DOCX) [file pntd.0005831.s003.docx]

**List of *Leptospira* spp. strains used for MAT panel**

| **ID** | **Species** | **Serogroup** | **Serovar** | **Strain** |
| --- | --- | --- | --- | --- |
| **S1** | *L. interrogans* | Djasiman | Djasiman | Djasiman |
| **S2** | *L. interrogans* | Bataviae | Bataviae | Swart |
| **S3** | *L. interrogans* | Pomona | Pomona | Pomona |
| **S4** | *L. borgpetersenii* | Mini | Mini | Sarl |
| **S5** | *L. noguchii* | Panama | Panama | CZ 214K |
| **S6** | *L. kirschneri* | Cynopteri | Cynopteri | 3522C |
| **S7** | *L. kirschneri* | Grippotyphosa | Grippotyphosa type Moskva | Moskva V |
| **S8** | *L. interrogans* | Pyrogenes | Pyrogenes | Salinem |
| **S9** | *L. interrogans* | Icterohaemorrhagiae | Copenhageni | Fiocruz L1-130 |
| **S10** | *L. interrogans* | Hebdomadis | Hebdomadis | Hebdomadis |
| **S11** | *L. interrogans* | Canicola | Canicola | Hond Utrecht IV |
| **S12** | *L. interrogans* | Autumnalis | Autumnalis | Akiyama A |
| **S13** | *L. interrogans* | Australis | Australis | Ballico |
| **S14** | *L. borgpetersenii* | Tarassovi | Tarassovi | Perepelitsin |
| **S15** | *L. borgpetersenii* | Sejroe | Sejroe | M84 |
| **S16** | *L. borgpetersenii* | Sejroe | Hardjo type Bovis | Sponselee |
| **S17** | *L. borgpetersenii* | Ballum | Castellonis | Castellon 3 |
| **S18** | *L. biflexa* | Semaranga | Patoc | Patoc I (Paris) |
| **S19** | *L. fainei* Hurstbridge/Hurstbridge (BUT 6^T^) | Hurstbridge | Hurstbridge | BUT 6^T^ |
| **S20** | *L. interrogans* | Icterohaemorrhagiae |  | Henriette* |

*****Reunion island local strain
