# Supplementary material for: Human leptospirosis in Seychelles: A prospective study confirms the heavy burden of the disease but suggests that rats are not the main reservoir
Source: PLoS Negl Trop Dis. 2017 Aug 28;11(8):e0005831. doi: 10.1371/journal.pntd.0005831 (PMC5591009; doi:10.1371/journal.pntd.0005831)
Supplement: S1 Checklist — (DOC) [file pntd.0005831.s005.doc]

STROBE Statement—checklist of items that should be included in reports of observational studies

|  | Item No | Recommendation | Done |
| --- | --- | --- | --- |
| **Title and abstract** | 1 | (*a*) Indicate the study’s design with a commonly used term in the title or the abstract | Line 1, 40 |
|  |  | (*b*) Provide in the abstract an informative and balanced summary of what was done and what was found | Line 39 - 50 |
| Introduction | | |  |
| Background/rationale | 2 | Explain the scientific background and rationale for the investigation being reported | Line 77 -132 |
| Objectives | 3 | State specific objectives, including any prespecified hypotheses | Line 132 - 137 |
| Methods | | |  |
| Study design | 4 | Present key elements of study design early in the paper | Line 153 -167 and Line 169 - 191 |
| Setting | 5 | Describe the setting, locations, and relevant dates, including periods of recruitment, exposure, follow-up, and data collection | Line 153 -167 and Line 169 - 191 |
| Participants | 6 | (*a*) *Cohort study*—Give the eligibility criteria, and the sources and methods of selection of participants. Describe methods of follow-up  *Case-control study*—Give the eligibility criteria, and the sources and methods of case ascertainment and control selection. Give the rationale for the choice of cases and controls  *Cross-sectional study*—Give the eligibility criteria, and the sources and methods of selection of participants | Line 157 -164. (Line 897) and Line 169 – 176. Box 1 and Figure 1. |
|  |  | (*b*) *Cohort study*—For matched studies, give matching criteria and number of exposed and unexposed  *Case-control study*—For matched studies, give matching criteria and the number of controls per case | NA |
| Variables | 7 | Clearly define all outcomes, exposures, predictors, potential confounders, and effect modifiers. Give diagnostic criteria, if applicable | **RT-PCR and MLST genotyping**  Line 219 – 247  **Leptospira Culture**  Line 248 -261  **Serology by ELISA and MAT**  Line 262 - 287  **Diagnostic Criteria**  Line 288 – 298  ***Rattus* variables**  Line 304 - 314 |
| Data sources/ measurement | 8* | For each variable of interest, give sources of data and details of methods of assessment (measurement). Describe comparability of assessment methods if there is more than one group | **RT-PCR and MLST genotyping**  Line 219 – 247  **Leptospira Culture**  Line 248 -261  **Serology by ELISA and MAT**  Line 262 - 287 |
| Bias | 9 | Describe any efforts to address potential sources of bias | Line 295 -298  “Samples that were positive for IgM ELISA only were considered negative due to the possibility of rheumatoid factors giving a false positivity and also for the well-known long-term persistence of anti-*Leptospira* IgM antibodies months and years after acute infection” |
| Study size | 10 | Explain how the study size was arrived at | Line 155 – 164 and Line 169 -178 |
| Quantitative variables | 11 | Explain how quantitative variables were handled in the analyses. If applicable, describe which groupings were chosen and why | Line 303 - 314 |
| Statistical methods | 12 | (*a*) Describe all statistical methods, including those used to control for confounding | Line 303 -314 |
|  |  | (*b*) Describe any methods used to examine subgroups and interactions | Line 311 - 314 |
|  |  | (*c*) Explain how missing data were addressed | NA |
|  |  | (*d*) *Cohort study*—If applicable, explain how loss to follow-up was addressed  *Case-control study*—If applicable, explain how matching of cases and controls was addressed  *Cross-sectional study*—If applicable, describe analytical methods taking account of sampling strategy | NA |
|  |  | (*e*) Describe any sensitivity analyses | NA |

Continued on next page

| Results |  | | |
| --- | --- | --- | --- |
| Participants 13* | (a) Report numbers of individuals at each stage of study—eg numbers potentially eligible, examined for eligibility, confirmed eligible, included in the study, completing follow-up, and analysed  (b) Give reasons for non-participation at each stage  (c) Consider use of a flow diagram |  | Human diseased (N=51), Human unaffected (N=172)  Line 322 – 329  *Rattus* spp. infected (N=57 or 7.7% of 739 *Rattus* spp. sampled)  Line 389 – 390  Line 322 - 325 |
|  |  |  |  |
|  |  |  | Line 340  Fig.1 Diagnostic flow chart of tests done, number of enrolled patients and diagnostic results |
| Descriptive data 14* | (a) Give characteristics of study participants (eg demographic, clinical, social) and information on exposures and potential confounders  (b) Indicate number of participants with missing data for each variable of interest  (c) *Cohort study*—Summarise follow-up time (eg, average and total amount) |  | Supplementary S1 Table and Supplementary S4 Table |
|  |  |  |  |
|  |  |  | Line 322 -325 (3 patients exluded, 1 refused participation and 2 did not provide sample to be tested  Line 341 - 346 |
| Outcome data 15* | *Cohort study*—Report numbers of outcome events or summary measures over time  *Case-control study—*Report numbers in each exposure category, or summary measures of exposure  *Cross-sectional study—*Report numbers of outcome events or summary measures |  | NA |
|  |  |  | NA |
|  |  |  | NA |
| Main results 16 | (*a*) Give unadjusted estimates and, if applicable, confounder-adjusted estimates and their precision (eg, 95% confidence interval). Make clear which confounders were adjusted for and why they were included  (*b*) Report category boundaries when continuous variables were categorized  (*c*) If relevant, consider translating estimates of relative risk into absolute risk for a meaningful time period |  | NA |
|  |  |  | NA |
|  |  |  | NA |
| Other analyses 17 | Report other analyses done—eg analyses of subgroups and interactions, and sensitivity analyses |  | **Genotyping by MLST**  Line 372 – 387, Line 397 – 403, and Line 478 – 480  **Biotic/Abiotic variable for *Rattus***  Line 412 - 470 |
| Discussion |  | | |
| Key results 18 | Summarise key results with reference to study objectives |  | **Line 132 – 134 Incidence**  Refer Line 482 – 490  **Line 134 – 136 Characterising *Leptospira* spp**  Refer Line 524 – 550  **Line 136 – 137 Identifying influences of biotic and abiotic factors**  Refer Line 551 - 608 |
| Limitations 19 | Discuss limitations of the study, taking into account sources of potential bias or imprecision. Discuss both direction and magnitude of any potential bias |  | Line 495 – 500  Line 502 – 515  Line 532 – 535  Line 548 – 550  Line 565 – 566  Line 590 – 592  Line 603 – 608  Line 616 – 619 |
| Interpretation 20 | Give a cautious overall interpretation of results considering objectives, limitations, multiplicity of analyses, results from similar studies, and other relevant evidence |  | Line 624 – 633 |
| Generalisability 21 | Discuss the generalisability (external validity) of the study results |  | Line 634 -647 |
| Other information |  | | |
| Funding 22 | Give the source of funding and the role of the funders for the present study and, if applicable, for the original study on which the present article is based |  | FEDER-POCT LeptOI #32913 and Agence Inter Etablissement de Recherche pour le Développement |

*Give information separately for cases and controls in case-control studies and, if applicable, for exposed and unexposed groups in cohort and cross-sectional studies.

**Note:** An Explanation and Elaboration article discusses each checklist item and gives methodological background and published examples of transparent reporting. The STROBE checklist is best used in conjunction with this article (freely available on the Web sites of PLoS Medicine at http://www.plosmedicine.org/, Annals of Internal Medicine at http://www.annals.org/, and Epidemiology at http://www.epidem.com/). Information on the STROBE Initiative is available at www.strobe-statement.org.
